# Upgrading right ventricular pacing to left bundle branch area pacing in pacemaker-induced cardiomyopathy: a systematic review and meta-analysis

**DOI:** 10.1186/s12872-026-06430-3

**Published:** 2026-08-14

**Authors:** Sami Shoura, Shreyas Singireddy, Ragavendar Saravanabavanandan, Abdulrahim Shilbayeh, Skylar Murphy, Chetan Bhardwaj, Timir Baman, Amjad AbuAlSuod

**Affiliations:** 1 Department of Cardiology, University of Illinois College of Medicine at Peoria, Peoria, Illinois USA; 2 Department of Medicine, University of Illinois College of Medicine at Peoria, Peoria, Illinois USA; 3 Department of Hospitalist Medicine, OSF Saint Francis Medical Center, Peoria, Illinois USA; 4 Department of Cardiology, Advanced Heart Failure Division, OSF Saint Francis Medical Center, Peoria, Illinois USA; 5 Department of Cardiology, Electrophysiology Division, OSF Saint Francis Medical Center, Peoria, Illinois USA

**Keywords:** Left bundle branch area pacing, Pacemaker-induced cardiomyopathy, Right ventricular pacing, Device upgrade, Cardiac resynchronization therapy, Systematic review

## Abstract

**Background:**

Chronic right ventricular pacing (RVP) causes pacemaker-induced cardiomyopathy (PICM) in 10–20% of patients. Left bundle branch area pacing (LBBAP) restores His-Purkinje activation and offers an alternative upgrade strategy; however, no formal pooled analysis comparing LBBAP versus biventricular pacing (BiVP) exists.

**Methods:**

We searched PubMed, Embase, Cochrane, and Scopus from inception to March 2026, and pooled effect sizes using restricted maximum likelihood (REML) methods. Nine studies contributed to the single-arm meta-analysis; one further study (Huang et al.) provided comparative LBBAP-versus-BiVP data only. Full database-specific search strategies are provided in Supplemental Text S1.

**Results:**

Nine observational studies (*N* = 242) were eligible. LBBAP succeeded in 96.4% of the procedures (95% CI 92.2–99.3%). Pooled left ventricular ejection fraction (LVEF) improved by 11.72% points (95% CI + 9.85 to + 13.59), paced QRS narrowed by 51.83 ms, left ventricular end diastolic diameter (LVEDD) decreased by 4.10 mm (k = 5), and NYHA improved by 0.98 points (all *p* < 0.001). Three non-fatal device-related complications were recorded across 242 patients (pooled rate 1.8%). In two observational head-to-head studies (*N* = 170) — one of which (Huang et al.) contributed comparative data only and is not among the nine single-arm studies — LBBAP was associated with a 4.59%-point greater LVEF improvement than BiVP (95% CI + 2.23 to + 6.95); with only two nonrandomized studies, this comparison is hypothesis-generating. Sensitivity analyses confirmed the robustness of the single-arm estimates.

**Conclusion:**

LBBAP upgrade was associated with improvement in LVEF and symptoms in patients with PICM. Limited observational data suggest greater LVEF improvement with LBBAP than BiVP, but this finding remains hypothesis-generating. Adequately powered randomized trials with hard clinical endpoints are needed.

**Trial registration:**

This review was registered in PROSPERO (CRD420261381943) after the search but before data extraction: Upgrading right ventricular pacing to left bundle branch area pacing in pacemaker-induced cardiomyopathy: a systematic review and meta-analysis.

**Graphical Abstract:**

**Figure:**
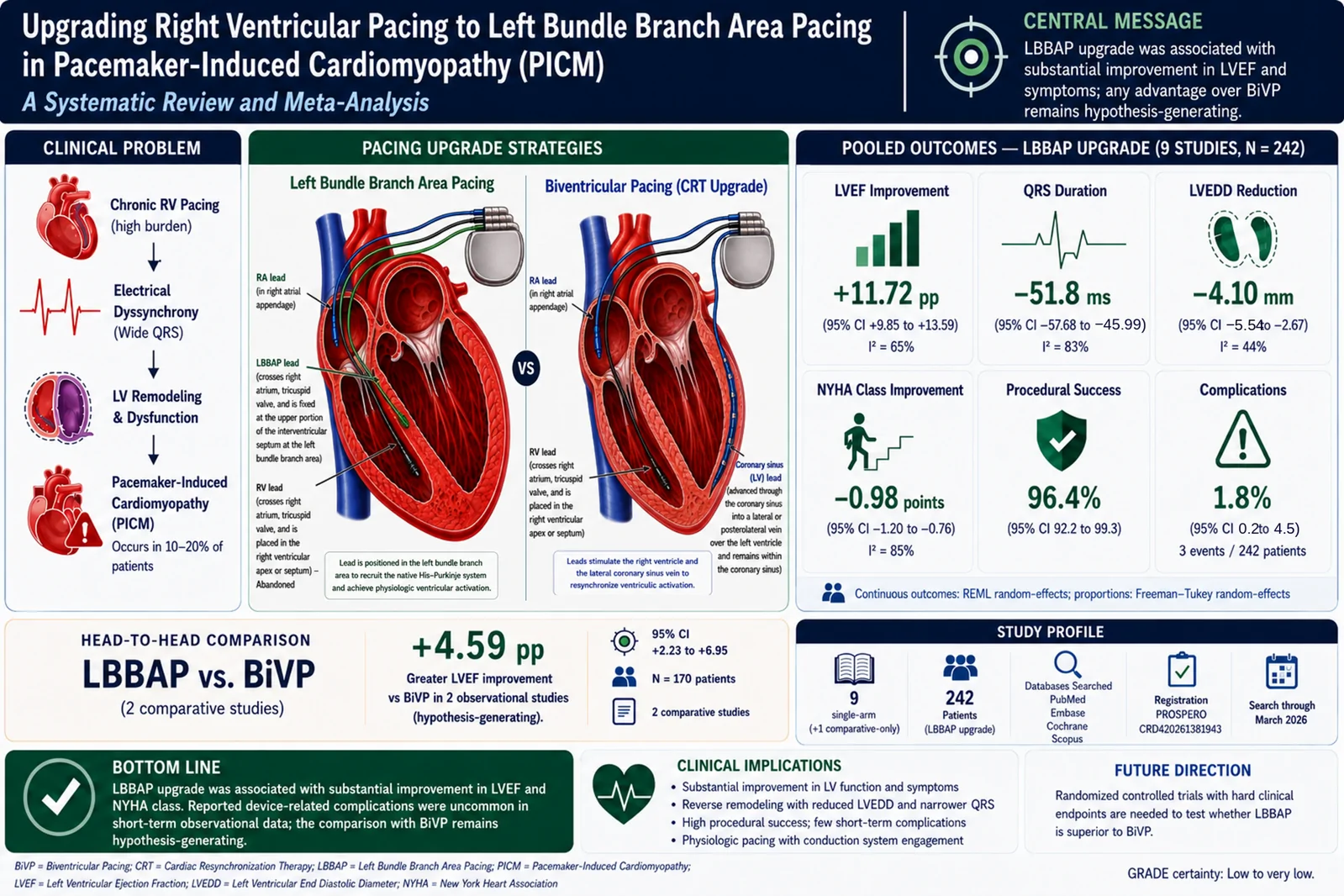

**Supplementary Information:**

The online version contains supplementary material available at https://doi.org/10.1186/s12872-026-06430-3.

## Capsule summary

In nine observational studies (n=242), left bundle branch area pacing (LBBAP) upgrade for pacemaker-induced cardiomyopathy succeeded in 96.4% of cases and improved the left ventricular ejection fraction (LVEF) by approximately 12 percentage points; head-to-head comparison suggested greater LVEF improvement with LBBAP than biventricular pacing, though this remains hypothesis-generating.

## Introduction

Right ventricular pacing (RVP) results in a dyssynchronous ventricular activation sequence resembling a left bundle branch block. If this phenomenon persists, left ventricular remodeling and systolic dysfunction can develop [1, 2]. Pacemaker-induced cardiomyopathy (PICM) develops in 10–20% of patients with a high RVP burden. It is defined by a ≥ 10%-point decline in the left ventricular ejection fraction (LVEF) to below 50% in the setting of an RVP burden > 20% in the absence of other causes of cardiomyopathy [3, 4]. Clinicians face a difficult choice when PICM develops. Options include upgrading the conventional RVP system to biventricular pacing (BiVP)-based cardiac resynchronization therapy (CRT) per current guidelines [5, 6] or to left bundle branch area pacing (LBBAP), which recruits the native conduction system distal to the typical site of the block. The strategy that yields greater functional recovery, with a lower risk profile, guides decision-making. Compared with BiVP, LBBAP provides a more favorable QRS duration in randomized de novo CRT populations [7–9]. Recent data from the LBBP-FAVOUR randomized trial, which focused on preventing RVP-induced cardiomyopathy, support physiologic pacing. The trial enrolled 160 patients with an anticipated high pacing burden and at least one cardiac vulnerability factor. The subjects were followed for a median of 36 months. The study revealed that LBBAP reduced the combined outcome of all-cause mortality, heart failure hospitalization (HFH), and PICM by approximately 69% (HR 0.31, 95% CI 0.145–0.664). These findings were attributed to a lower incidence of PICM in the LBBAP group (HR 0.324, 95% CI 0.119–0.883) [10]. LBBP-FAVOUR reported that LBBAP prevents progression to PICM but does not demonstrate the ability of LBBAP to reverse established ventricular dysfunction. Within conduction system pacing, His bundle pacing has been progressively supplanted by LBBAP in contemporary practice owing to higher and less stable capture thresholds, greater lead-revision rates, and technical demands so pooling the two obscures the effect of the strategy clinicians now actually use. Prior reviews either combined HBP and LBBAP, ended their searches before LBBAP data became available (2024–2026), or did not compare LBBAP with BiVP. Our systematic review and meta-analysis address these gaps. We restricted the selection to LBBAP and searched from database inception through March 2026. For the first time, we pooled head-to-head LBBAP-versus-BiVP upgrade data as a formal primary outcome.

## Methods

### Protocol registration

This systematic review and meta-analysis was conducted in accordance with the PRISMA 2020 statement [11]. The review protocol was registered in PROSPERO (CRD420261381943) after the search was completed but before data extraction. Four deviations from the registered protocol are disclosed per PRISMA 2020 item 24c. First, restricted maximum likelihood (REML) was used as the primary estimator of heterogeneity instead of the DerSimonian–Laird (DL) method. Second, the methodological index for nonrandomized studies (MINORS) was selected for risk-of-bias assessment, given the mixed comparative and noncomparative observational designs in the evidence base [12]. Third, three prespecified subgroup analyses (stratified by infranodal atrioventricular block, true LBBAP versus LV septal pacing, and baseline LVEF severity) were not performed because the final study count (k = 9) was insufficient for reliable covariate adjustment. Fourth, two studies (Moraleda-Salas et al. [13] and Shah et al. [14]) with follow-up durations slightly outside the registered 6–24 month period (4.7 months and 27.3 months, respectively) were retained because both reported the registered outcomes with intact pre- and post-upgrade data. None of these deviations altered the registered PICO framework, primary outcomes, or eligibility criteria. Finally, 6 additional authors were added.

### Search strategy

A systematic literature search was conducted from inception through March 31, 2026, across four electronic databases: PubMed (MEDLINE), Embase, the Cochrane Library (including CENTRAL), and Scopus. The search terms combined medical subject headings (MeSH) and free-text keywords across three conceptual domains were as follows: (1) pacing modality- “left bundle branch pacing”, “left bundle branch area pacing”, “LBBP”, “LBBAP”, “conduction system pacing”; (2) clinical context- “right ventricular pacing”, “pacemaker upgrade”, “device upgrade”, “pacing-induced cardiomyopathy”, “PICM”; and (3) intervention or outcome- “upgrade”, “cardiac resynchronization”. To supplement the electronic database search, backward citation and gray literature searches were also conducted. The gray literature sources included conference abstracts and proceedings from the Heart Rhythm Society, the European Society of Cardiology, and the American College of Cardiology (2020–2025) and trial registries, including ClinicalTrials.gov and the ISRCTN registry. Full database-specific search strings, including field tags and date limits for each database, are provided in Supplemental Text S1.

### Eligibility criteria

Eligible studies included adults aged 18 years or older with PICM or clinically significant left ventricular dysfunction attributable to a chronic right ventricular pacing burden of 20% or greater who were undergoing device upgrade to LBBAP. The comparator was either a pre-upgrade baseline in single-arm studies or BiVP in comparative studies. The primary outcomes were the LBBAP procedural success rate and the change in the left ventricular ejection fraction (LVEF). The secondary outcomes were QRS duration reduction, left ventricular end-diastolic diameter (LVEDD) reduction, NYHA functional class improvement, heart failure hospitalization, all-cause mortality, and the procedural complication rate. The comparative outcome was the between-group mean difference in LVEF improvement between the LBBAP and BiVP upgrade arms. Studies were excluded if they (1) reported His bundle pacing exclusively without extractable LBBAP outcomes, (2) were case series with fewer than 10 LBBAP patients, (3) enrolled de novo cardiac resynchronization therapy recipients without a chronic right ventricular pacing background, (4) were conference abstracts without accessible full data, or (5) were prior systematic reviews or meta-analyses to avoid double-counting of primary study patients.

### Data extraction and risk of bias

Two reviewers independently extracted data through a standardized template. The extracted variables included study design, patient demographics, right ventricular pacing duration and burden, baseline and follow-up measurements of the LVEF, QRS duration, LVEDD, and NYHA functional class, procedural success rates, pacing parameters, complication rates, and the proportion of true left bundle branch capture versus left ventricular septal pacing [15], where reported. Discrepancies between reviewers were resolved by discussion and consensus.

The risk of bias was assessed using the MINORS tool, selected for its suitability across both comparative and noncomparative observational designs. Noncomparative studies were rated as high quality (scores of 12 or above), moderate quality (9–11), or low quality (below 9). Comparative studies were rated as high quality (scores of 18 or above), moderate quality (14–17), or low quality (below 14). The certainty of evidence was assessed via the Grading of Recommendations, Assessment, Development, and Evaluations (GRADE) framework.

### Statistical analysis

Pooled mean differences (MDs) with 95% confidence intervals (CIs) were computed via a random-effects model with restricted maximum likelihood (REML) estimation of between-study variance (τ²). REML was selected over the DerSimonian–Laird (DL) estimator because it produces less biased τ² estimates when the number of contributing studies is small to moderate and represents current best-practice guidance from the Cochrane Collaboration [16]. DerSimonian–Laird estimates were computed in parallel as an independent validation. The REML implementation was validated against a published benchmark dataset prior to application. When the change-score standard deviation was directly reported by the source study, the reported value was used. When only pre- and post-upgrade means and standard deviations were available, the change-score standard deviation was estimated as SDchange = √(SDpre² + SDpost² − 2r·SDpre·SDpost), assuming a within-subject correlation of *r* = 0.5, which is consistent with the Cochrane Handbook recommendations. A prespecified sensitivity analysis was performed across *r* = 0.2 to 0.8, in increments of 0.1, for the primary LVEF outcome to assess robustness to this assumption. Five of nine studies reported change-score standard deviations directly and were unaffected by the correlation assumption, the sensitivity analysis, therefore, reflects the influence of r on the four remaining studies in which the SDchange was estimated.

Pooled proportions, specifically the procedural success rate and complication rate, were estimated via Freeman–Tukey double arcsine transformation with REML estimation of τ² on the transformed scale and back-transformed via the harmonic mean sample size [17]. This approach was selected to stabilize the variance for proportions approaching 0% or 100%, as encountered in the procedural success data.

Heterogeneity was quantified via Cochran’s Q statistic and the I² statistic, interpreted as low (< 25%), moderate (25–50%), or high (> 50%). Meta-regression was not performed given the insufficient study count for reliable covariate adjustment (k = 9). Leave-one-out sensitivity analyses were conducted for all primary and secondary outcomes. Funnel plot asymmetry was assessed visually and formally via Egger’s regression intercept test. The trim-and-fill method was not applied because of the limited statistical power of asymmetry tests, with fewer than 10 studies. All tests were two-sided, and p-values less than 0.05 were considered statistically significant. Analyses were performed in R (version 4.3) using the Metafor package.

### Use of artificial intelligence

Artificial intelligence tools were used in a limited and supplementary capacity during manuscript preparation. Grammarly and Curie were used to refine the grammar and language. All scientific decisions, including study selection, data extraction, source paper verification, statistical analysis, and interpretation of findings, were made exclusively by the authors. ChatGPT was used to create the graphical abstract figure. The authors accept full responsibility for the integrity and accuracy of the reported work.

## Results

### Study selection

**Fig. 1:**
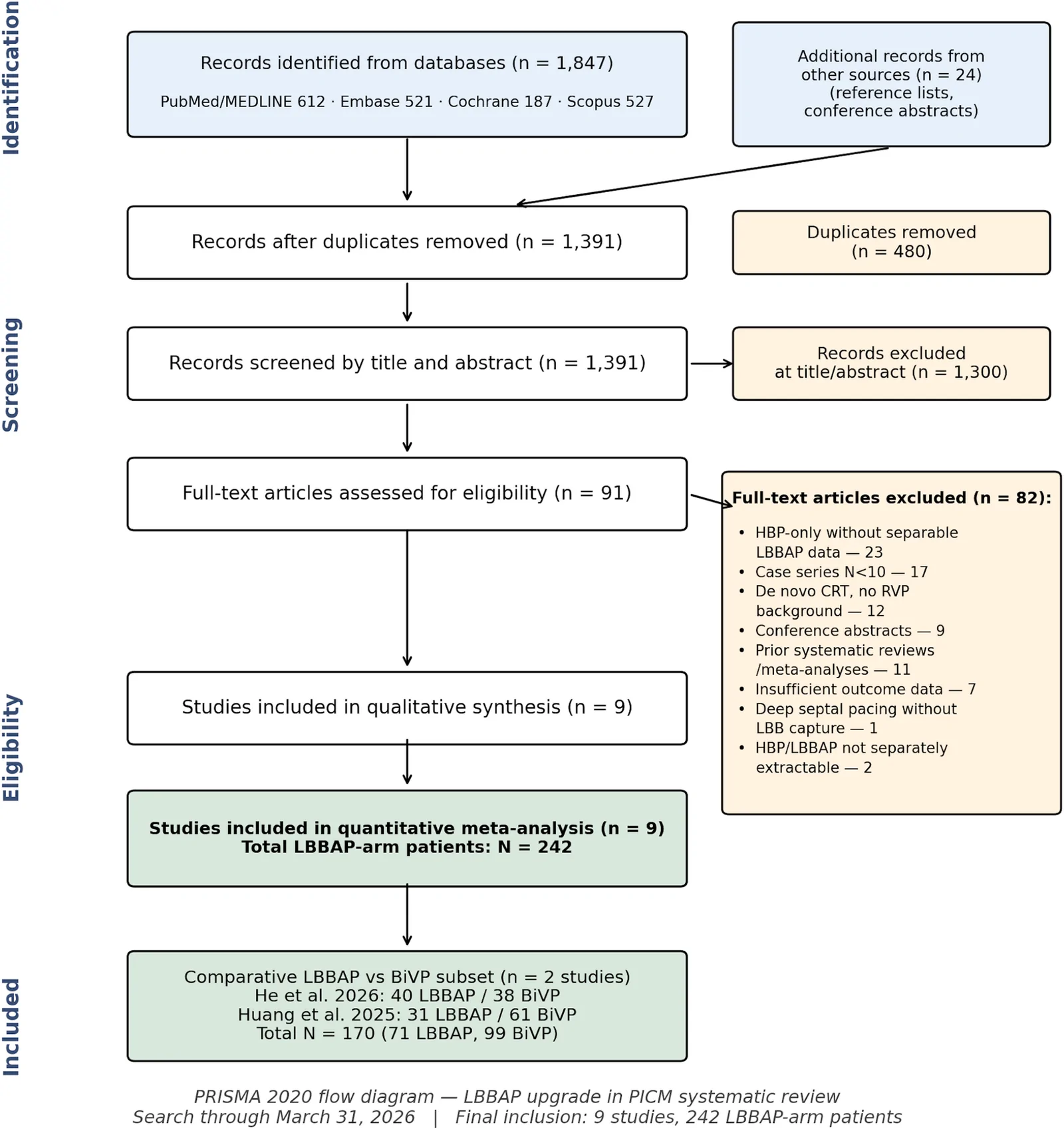
PRISMA 2020 flow diagram. Nine primary studies (*N* = 242 LBBAP-arm patients) met all eligibility criteria. The included tier additionally shows the comparative subset (*n* = 2 head-to-head LBBAP-versus-BiVP studies, total *N* = 170). Huang et al. contributed comparative data only and is not counted among the nine single-arm studies

The database search identified 1,847 records, and backward and gray literature searches added 24 records, for a total of 1,871. After removing 480 duplicates, 1,391 records were screened for titles and abstracts. Ninety-one full-text articles were assessed for eligibility, 82 of which were excluded. The reasons for exclusion included HBP-only studies without separable LBBAP data (*n* = 23), case series with fewer than 10 LBBAP patients (*n* = 17), de novo CRT recipients without a chronic RVP background (*n* = 12), prior systematic reviews or meta-analyses (*n* = 11), conference abstracts without accessible full data (*n* = 9), insufficient outcome data (*n* = 7), deep septal pacing without confirmed left bundle branch capture (*n* = 1), and mixed HBP and LBBAP cohorts in which arm-specific data were not separately extractable (*n* = 2). Nine primary studies, comprising 242 LBBAP-arm patients, met all eligibility criteria and were included in the meta-analysis. The PRISMA 2020 flow diagram is presented in Fig. 1. One additional study (Huang et al.) provided comparative LBBAP-versus-BiVP data only and was not counted among the nine single-arm studies.

### Characteristics of the included studies

The nine included studies were published between 2021 and 2026 and are summarized in Table 1. Four were prospective and five were retrospective. All studies were single-center, except He et al., [18] which was conducted across two centers, and Shah et al., which was conducted across three centers. The LBBAP-arm sample sizes ranged from 10 (Moraleda-Salas et al.) to 64 (Shah et al.). The mean baseline LVEF ranged from 31% to 40.3% across studies, and the right ventricular pacing burden exceeded 90% in all studies that reported this parameter. The follow-up duration ranged from 4.7 months (Moraleda-Salas et al.) to 27.3 months (Shah et al.). Five of the nine studies originated from Chinese centers — Ye et al. [19], Qian et al. [20], Shan et al. [21], He et al. [18], and Pei-pei M et al. [22] and potential patient overlap across these cohorts cannot be excluded, as acknowledged in the limitations. One of the studies was conducted in Netherlands [23]. Study-level PICM definitions, baseline RVP burden, and available LBBAP capture data are summarized in Supplemental Table S7. Most included studies reported outcomes for LBBAP as a single group rather than separating true left bundle branch pacing (LBBP) from left ventricular septal pacing (LVSP): only He et al. reported an explicit split (30 LBBP, 10 LVSP), whereas Ye et al. and Pei-pei M et al. analyzed only confirmed true-LBBP patients, and the remaining studies did not quantify the split. This variability in capture confirmation and subtype reporting may contribute to clinical heterogeneity in pooled efficacy.

**Table 1:** Characteristics of the included primary studies—LBBAP upgrade from chronic RVP

Study (Year)	Country, Design	*N*	Age, y	Male, %	RVP burden, %	FU, mo	Success, *n*/*N*	MINORS
Rademakers 2022 [23]	Netherlands, Prospective	20	75	70	99 ± 1.5	6	20/20	14/16
Ye 2021 [19]	China, Retrospective	19	67.6	78.9	99 ± 1.5	13.5 ± 6.2	19/19	12/16
Qian 2021 [20]	China, Prospective	13	70	76.9	96.5 ± 7.0	10.4 ± 6.1	13/13	14/16
Perea-Armijo 2025 [24]	Spain, Prospective	25	73	72	98.5 [97–99]	18.5 (med)	21/25	14/16
Pei-pei M 2024 [22] †	China, Retrospective	13	71	70	~ 93	27.8 ± 9.7	13/13	20/24
Shan 2023 [21]	China, Retrospective	38	68.4	71.1	97.9 ± 3.7	12	34/38	12/16
He 2026 [18] ‡	China, Prospective	40	70.8	67.5	92.9 ± 13.6	20.3 ± 12.4	40/40	22/24
Shah 2026 [14]	USA, Retrospective.	64	71	70	95.4 ± 10.4	27.3 ± 16.5	64/64	13/16
Moraleda-Salas 2025 [13] §	Spain, Prospective.	10	75	60	~ 100	4.7 (med)	10/10	11/16

Values are mean ± SD unless noted; [ ] = median [IQR]; (med) = median. MINORS quality: high ≥ 12/16 (non-comparative) or ≥ 18/24 (comparative); all studies were high quality except Moraleda-Salas (moderate)

*FU *follow-up, *RVP *right ventricular pacing

†Pei-pei M: LBBP arm (*N*=13) extracted from a conduction-system-pacing cohort with an HBP comparator (MINORS scored /24)

‡He: comparative study with a BiVP comparator (*n*=38); single-arm LBBAP data are shown here (MINORS scored /24)

§Moraleda-Salas: LBBAP arm (*N*=10) extracted from a mixed HBP/LBBAP cohort

The risk of bias, assessed via MINORS, ranged from 11 to 22. Eight of the nine studies were rated high quality. Moraleda-Salas et al. reported moderate quality (11 of 16) owing to its short follow-up duration and small LBBAP-arm sample size. No study was rated low quality. Certainty of evidence, assessed via GRADE, ranged from low to very low across all outcomes, reflecting the nonrandomized observational design of the included studies; full GRADE ratings are presented in Table 2.

**Table 2:** GRADE certainty of evidence summary

Outcome	Design	k	Risk of bias	Inconsistency	Indirectness	Imprecision	Certainty (GRADE)
LVEF improvement (single-arm)	Observational	9	Serious	Moderate (I²=65%); not downgraded	Not serious	Not serious	⊕⊕○○ LOW
QRS reduction	Observational	9	Serious	Very serious (I²=83%); downgraded	Not serious	Not serious	⊕○○○ VERY LOW
NYHA improvement	Observational	7	Serious	Very serious (I²=85%); downgraded	Not serious	Not serious	⊕○○○ VERY LOW
LVEDD reduction	Observational	5	Serious	Moderate (I²=44%)	Not serious	Not serious	⊕⊕○○ LOW
LBBAP vs. BiVP: LVEF (comparative)	Observational	2	Serious	None (I²=0%; uninformative)	Not serious	Serious (k = 2)	⊕○○○ VERY LOW

All outcomes derive from nonrandomized observational studies and therefore begin at low certainty. QRS reduction and NYHA improvement were each downgraded one further level for very high heterogeneity (I²>75%), yielding very low certainty. The comparative LVEF estimate was downgraded for imprecision because only two studies contributed data; an I²=0% based on two studies is not informative about true homogeneity. For single-arm LVEF, the moderate heterogeneity (I²=65%) did not warrant a further downgrade because the direction of effect was consistent across all nine studies, leave-one-out estimates clustered tightly (+ 11.22 to + 12.11%), and the r-sensitivity range was narrow (+ 11.59 to + 11.82%). Symbols: ⊕⊕○○ = low certainty; ⊕○○○ = very low certainty

### Primary outcomes

#### LVEF improvement

**Fig. 2:**
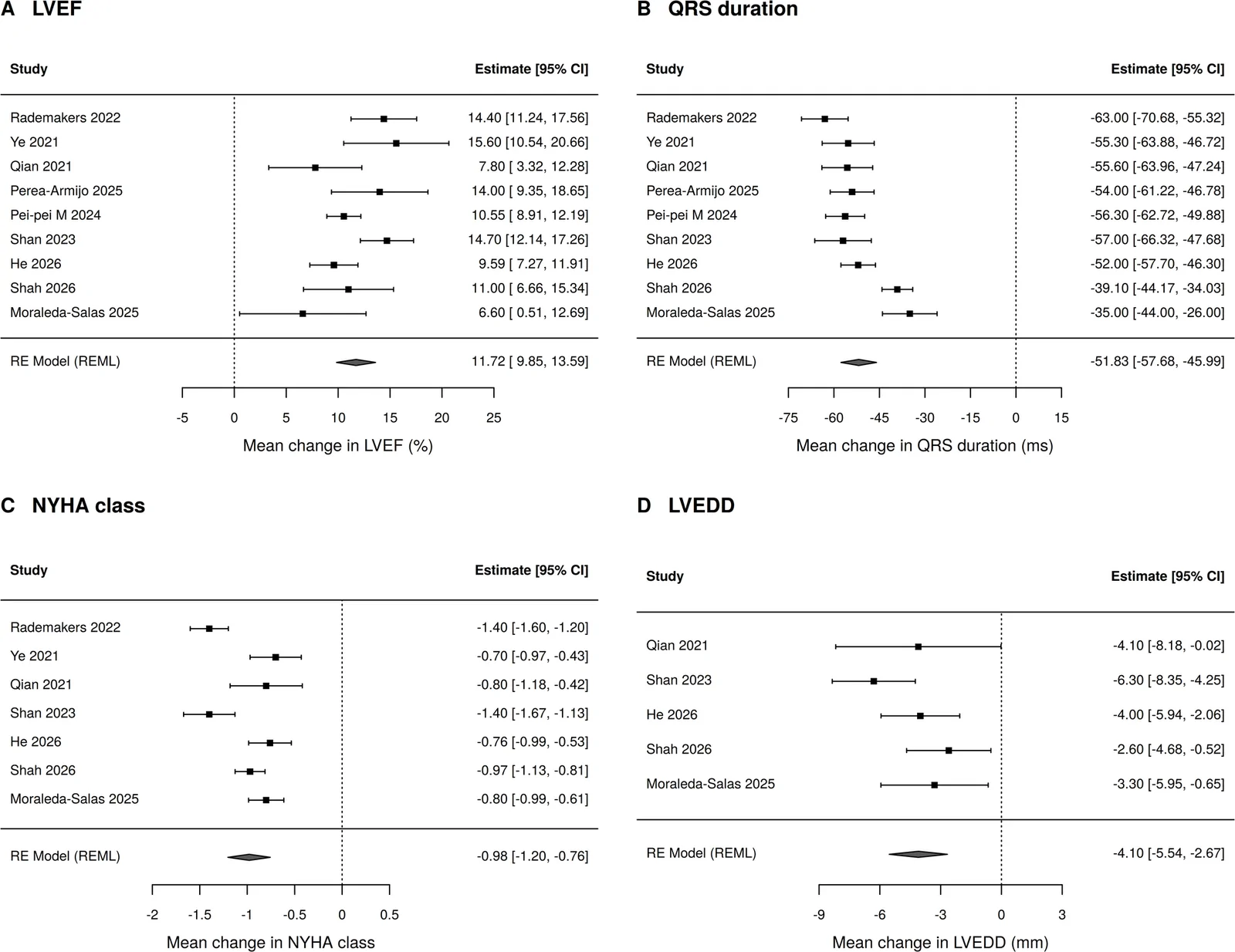
Forest plots of pooled continuous outcomes after upgrade to LBBAP. Random-effects (REML) pooled mean changes in (**A**) LVEF, (**B**) QRS duration, (**C**) NYHA functional class, and (**D**) LVEDD

The pooled LVEF improvement after the LBBAP upgrade was + 11.72% points (95% CI + 9.85 to + 13.59; *p* < 0.001; I²=65%; τ²=4.75; k = 9, *N* = 242). The direction of effect was consistent across all nine studies, with individual study estimates ranging from + 6.6% points (Moraleda-Salas et al. [13], median follow-up 4.7 months) to + 15.6% points (Ye et al. [19], mean follow-up 13.5 months). The full results are presented in Fig. 2 and Table 3.

**Table 3:** Pooled outcome estimates—LBBAP upgrade from chronic RVP (REML random effects)

Outcome	k	*N* (LBBAP)	Pooled Estimate (95% CI)	*p* value; I²; τ²
Procedural success rate (%)	9	242	96.4% (92.2–99.3%)	*p* < 0.001; I²=54%; τ²=0.010
LVEF improvement (MD, %)	9	242	+ 11.72% (+ 9.85 to + 13.59%)	*p* < 0.001; I²=65%; τ²=4.75
QRS duration reduction (MD, ms)‡	9	242	−51.83 ms (− 57.68 to − 45.99)	*p* < 0.001; I²=83%; τ²=65.23
LVEDD reduction (MD, mm)§	5	165	−4.10 mm (− 5.54 to − 2.67)	*p* < 0.001; I²=44%; τ²=1.15
NYHA class improvement (MD)†	7	204	−0.98 (− 1.20 to − 0.76)	*p* < 0.001; I²=85%; τ²=0.075
LVEF: LBBAP vs. BiVP (MD, %)*	2	170 (71/99)	+ 4.59% (+ 2.23 to + 6.95%) favoring LBBAP	*p* < 0.001; I²=0%; τ²=0
Clinical events (BiVP vs. LBBAP)	1	78 (He et al.)	HR 1.57 (0.55–4.48) for BiVP vs. LBBAP; 26.3% vs. 17.5% events; underpowered	*p* = 0.395 (NS)
Complication rate	9	242	1.8% (95% CI 0.2–4.5%) ; 3 events in 242 patients	I²=0%

*CI *confidence interval, *I² *heterogeneity, *τ² *between-study variance, *LBBAP *left bundle branch area pacing, *LVEDD *left ventricular end-diastolic diameter, *LVEF *left ventricular ejection fraction, *MD *mean difference, *NS *not significant

*Hypothesis-generating: based on two observational studies only. I²=0% with k=2 is statistically uninformative

†The elevated I² for NYHA indicates baseline distribution differences; leave‑one‑out analysis produced estimates between −0.91 and −1.02

‡High I² for the QRS complex indicates variability among patients in the baseline RVP QRS complex (158–193 ms); leave-one-out analysis consistently revealed estimates between −50.5 and −53.7 ms in every iteration

§Following an audit, the LVEDD was available in five studies (*N*=165). Ye 2021 reported the LVESV and was excluded from the analysis. Shah 2026 reported that the LVEDD (pre 53.9±8.0 → post 51.3±8.9 mm) was therefore included. The audit corrected a previous extraction error. All LVEF sensitivity analyses (r=0.2–0.8) produced consistent estimates (+11.59 to +11.82%) and p<0.001

#### Procedural success rate

**Fig. 3:**
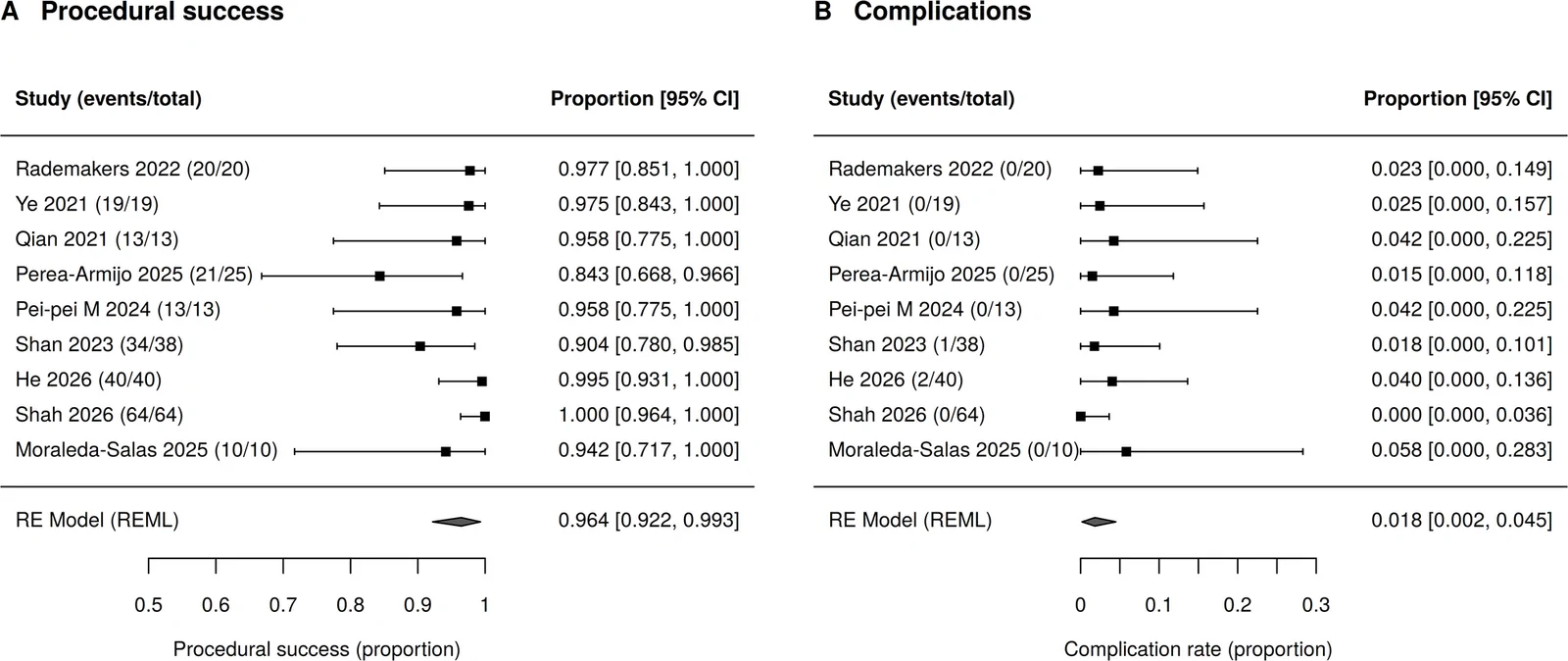
Forest plots of pooled procedural outcomes. Freeman–Tukey double-arcsine random-effects (REML) pooled proportions for (**A**) procedural success and (**B**) complications, with per-study event counts shown beside each study

LBBAP upgrades were successfully performed in 96.4% of the procedures (95% CI 92.2–99.3%; k = 9, *N* = 242; I²=54%; τ²=0.010). Five of nine studies reported a 100% success rate. The four remaining studies reported rates ranging from 84% to 98%. The lowest individual success rate was 84%, reported by Perea-Armijo et al. [24], in a cohort restricted to severe PICM with a baseline LVEF ≤ 40%. Procedural success and complication rates across studies are presented in Fig. 3.

### Secondary outcomes

#### QRS duration reduction

Paced QRS duration was reduced by a pooled mean of 51.83 ms (95% CI − 57.68 to − 45.99; *p* < 0.001; I²=83%; τ²=65.23; k = 9, *N* = 242) following the LBBAP upgrade. Post-upgrade paced QRS durations ranged from 114 to 135 ms across studies. Leave-one-out sensitivity analyses produced pooled estimates ranging from − 50.5 to − 53.7 ms across all iterations (Supplemental Table S2). The full results are presented in Table 3.

#### LVEDD reduction

The left ventricular end-diastolic diameter was reduced by a pooled mean of 4.10 mm (95% CI − 5.54 to − 2.67; *p* < 0.001; I²=44%; τ²=1.15; k = 5, *N* = 165) following the LBBAP upgrade. The full results are presented in Table 3.

#### NYHA functional class improvement

The NYHA functional class improved by a pooled mean of 0.98 points (95% CI − 1.20 to − 0.76; *p* < 0.001; I²=85%; τ²=0.075; k = 7, *N* = 204) following the LBBAP upgrade. Leave-one-out sensitivity analyses produced pooled estimates ranging from − 0.91 to − 1.02 across all iterations (Supplemental Table S3). The full results are presented in Table 3.

### Procedural safety

The pooled complication rate was 1.8% (95% CI 0.2–4.5%; I²=0%; k = 9, *N* = 242), corresponding to three device-related events across 242 patients. He et al. reported a pocket infection and an increased pacing threshold. Shan et al. reported one pocket hematoma in the PICM arm. No procedural deaths were reported across any included study. The full results are presented in Fig. 3 and Table 3.

### Sensitivity analysis

**Fig. 4:**
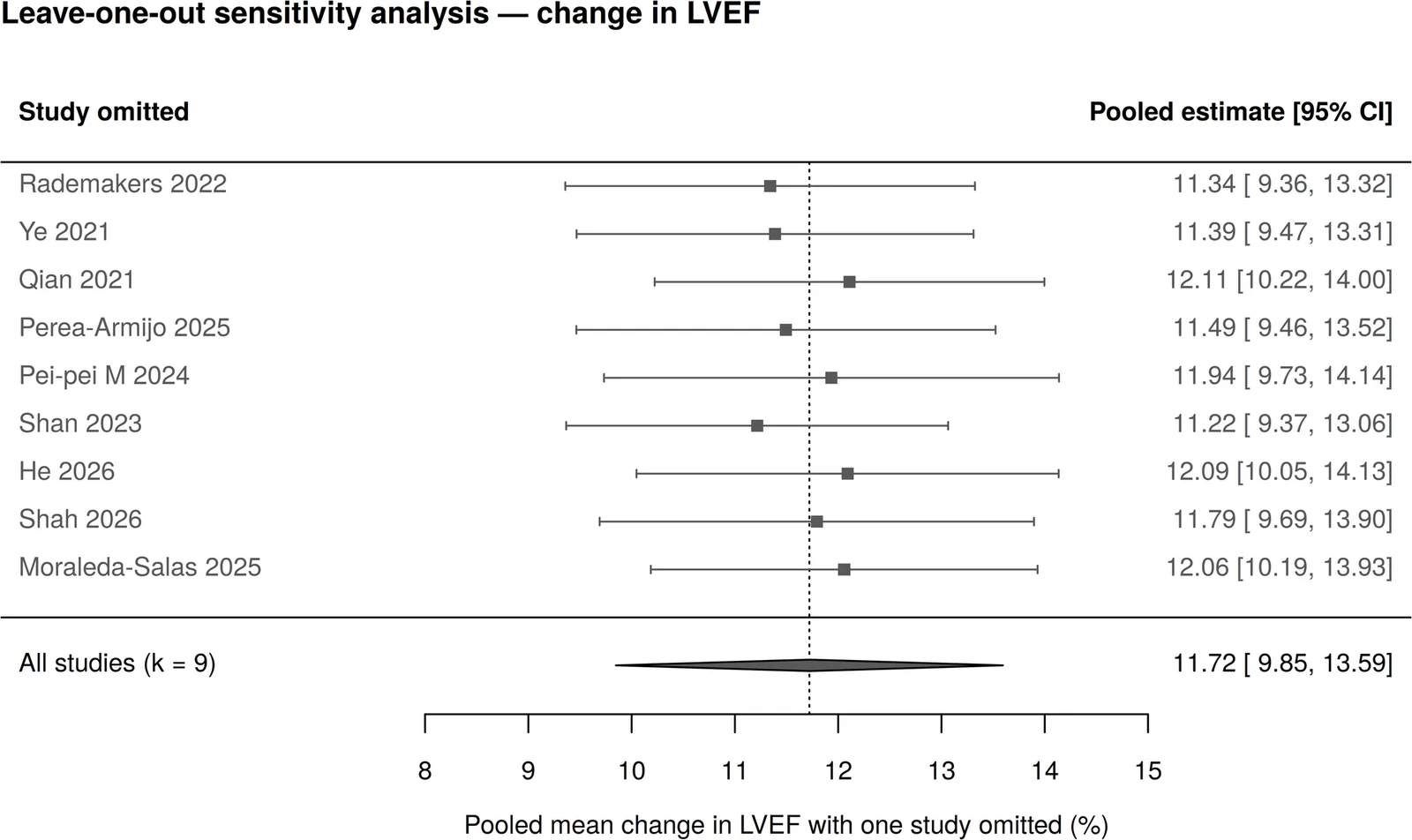
Leave-one-out sensitivity analysis for LVEF improvement. Each row shows the pooled estimate recomputed with the indicated study omitted; the dotted line marks the full-model estimate (+11.72 percentage points). Estimates remained between +11.22 and +12.11 across all iterations

**Fig. 5:**
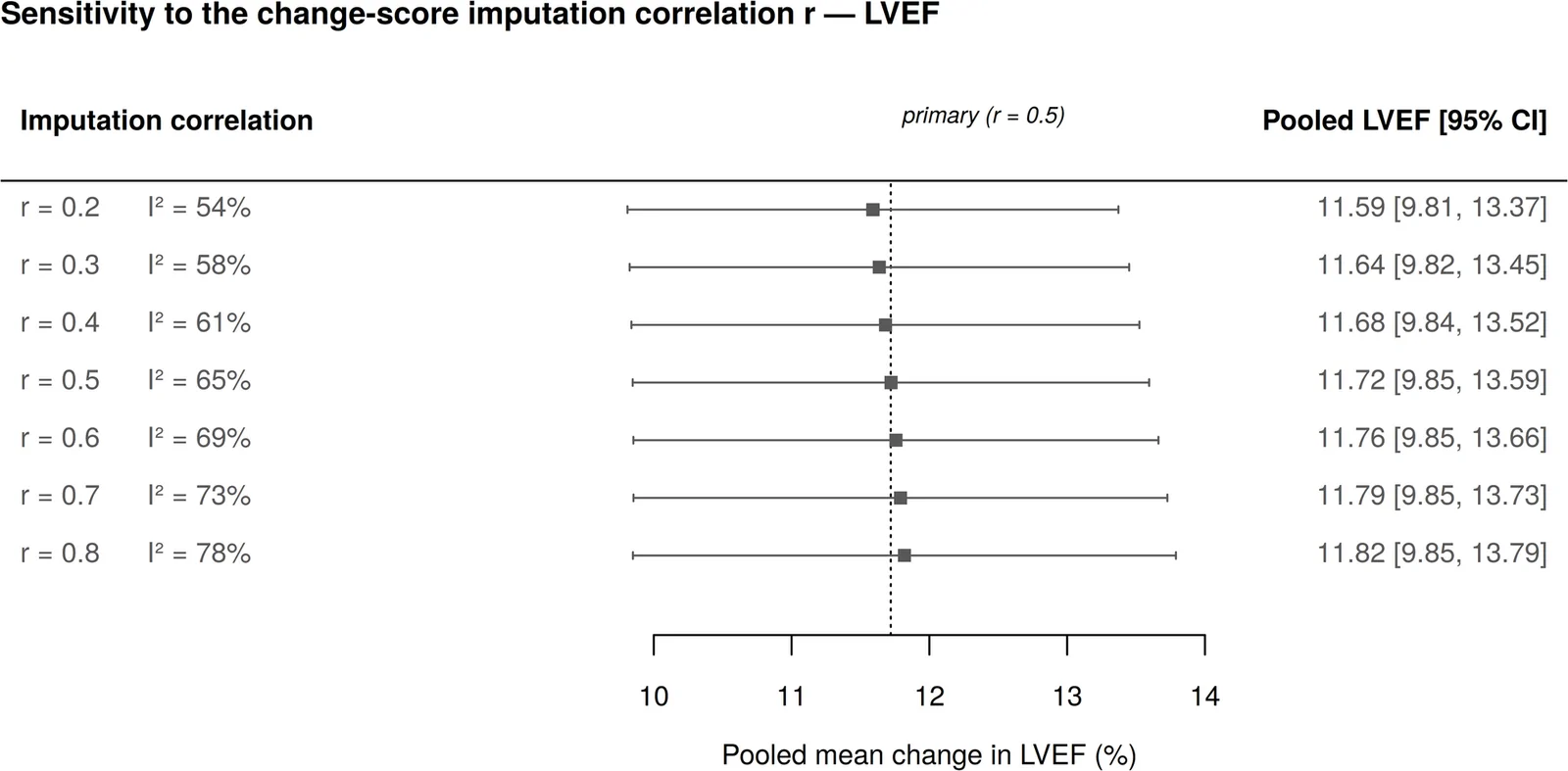
Sensitivity of the pooled LVEF estimate to the assumed change-score imputation correlation (r). Pooled estimates across r = 0.2–0.8 are shown; the dotted line marks the primary analysis (r = 0.5), and I² is reported for each value of r

The exclusion of Moraleda-Salas et al. — the study with the smallest LBBAP-arm sample size (*n* = 10) and shortest follow-up duration (4.7 months) — produced a pooled LVEF improvement of + 12.06% (95% CI + 10.19 to + 13.93%) across eight studies (*N* = 232), compared with + 11.72% in the primary analysis. The results for QRS duration, NYHA functional class, and LVEDD were similarly insensitive to this exclusion. Leave-one-out sensitivity analyses produced pooled estimates ranging from + 11.22 to + 12.11% points across all iterations (Fig. 4; Supplemental Table S1). The prespecified sensitivity analysis across *r* = 0.2 to 0.8 produced pooled estimates ranging from + 11.59% to + 11.82% (span 0.23% points), with I² values ranging from 53.9% to 77.9% across the same range (Fig. 5; Supplemental Table S4).

### Publication bias and small-study effects

**Fig. 6:**
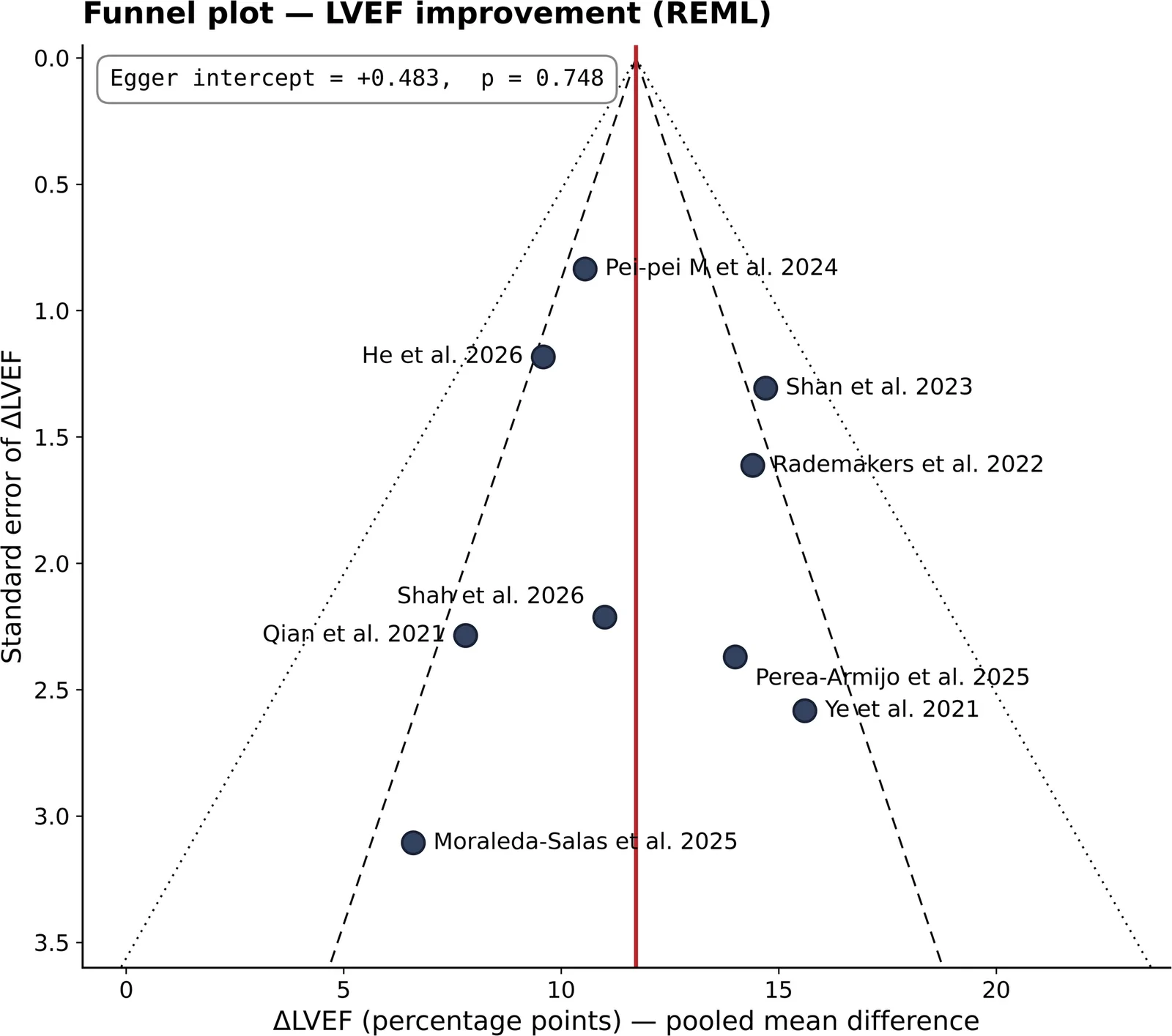
Funnel plot for assessment of publication bias and small-study effects (LVEF improvement). The standard error is plotted against the effect estimate; the red line marks the pooled estimate, and the dashed and dotted lines denote the 95% and 99.9% pseudo-confidence bounds. Egger’s regression test was nonsignificant (*p* = 0.75)

Funnel plot inspection revealed broadly symmetric dispersion of effect sizes around the pooled LVEF estimate (Fig. 6). Egger’s regression intercept test was nonsignificant (intercept = + 0.483; *p* = 0.748). Small-study effects cannot be definitively excluded, and formal asymmetry (publication bias) testing is underpowered with fewer than 10 studies.

### Comparative analysis: LBBAP versus biventricular pacing

Two studies directly compared LBBAP and BiVP upgrade strategies in patients with established PICM — He et al. and Huang et al. contributing a combined cohort of 170 patients (71 LBBAP, 99 BiVP). He et al. prospectively compared 40 LBBAP patients with 38 BiVP patients across two centers and reported greater LVEF improvement with LBBAP (+ 9.59 ± 7.48% versus + 4.91 ± 7.73%; *p* = 0.008). Clinical event rates were not significantly different between arms (17.5% LBBAP versus 26.3% BiVP; adjusted HR 1.57 for BiVP versus LBBAP, 95% CI 0.55–4.48; *p* = 0.395). Huang et al. retrospectively compared 31 LBBAP patients and 61 BiVP patients and reported greater LVEF improvement with LBBAP (+ 7.0 ± 7.0% versus + 2.5 ± 8.7%; *p* = 0.024), a shorter paced QRS duration (145.9 ± 22.4 ms versus 157.5 ± 26.5 ms; *p* = 0.031), and higher echocardiographic response rates (67.7% versus 39.3%; *p* = 0.019).

**Fig. 7:**
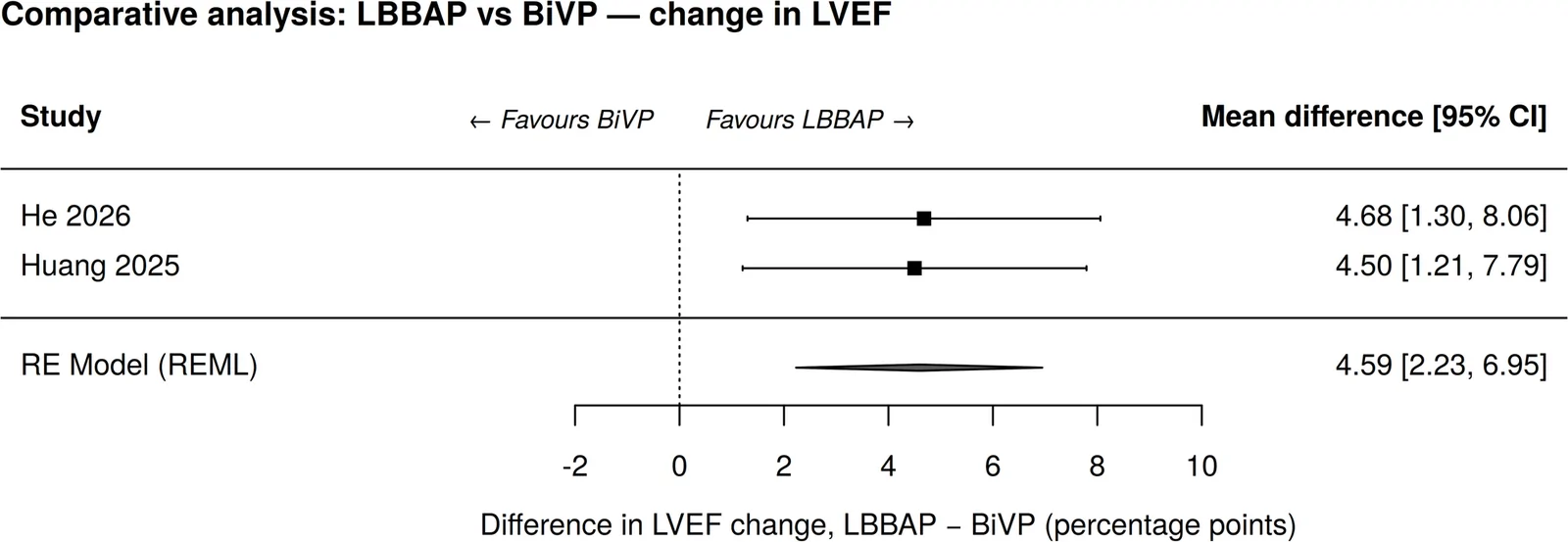
Comparative forest plot of LBBAP versus biventricular pacing (change in LVEF). Random-effects (REML) pooled between-group mean difference; positive values favor LBBAP. Based on two studies (k = 2; *N* = 170)

The pooled between-group mean difference in LVEF improvement favored LBBAP by 4.59% points (95% CI + 2.23 to + 6.95; *p* < 0.001; I²=0%; τ²=0; k = 2, *N* = 170). An I² of 0% based on two studies is not informative about true between-study homogeneity. Because Huang et al. did not report pre-upgrade LVEF separately for the LBBAP arm, that cohort was included in the comparative analysis only and excluded from the nine single-arm studies in Table 1. The full results are presented in Table 3 and Fig. 7.

## Discussion

This meta-analysis of nine observational studies enrolling 242 patients demonstrated that LBBAP upgrades are associated with meaningful improvement in ventricular function and symptoms in patients with PICM. LBBAP succeeded in 96.4% of the procedures and was associated with an average improvement in LVEF of approximately 12% points, a reduction in paced QRS duration of approximately 52 ms, a reduction in LVEDD of approximately 4 mm, and an improvement in the NYHA functional class by approximately one grade. The direction of effect was consistent across all included studies. In the two studies that directly compared LBBAP with the BiVP upgrade, LBBAP was associated with approximately 4.6% points greater LVEF recovery. Complications were uncommon, occurring in 1.8% of procedures, with no procedural deaths reported. Certainty of evidence was rated low to very low by GRADE, reflecting the nonrandomized observational design of all included studies.

### Mechanisms of ventricular function improvement after LBBAP upgrade

Right ventricular pacing imposes a wide, dysynchronous activation sequence that propagates through the myocardium rather than through the native conduction system, resembling left bundle branch block morphology [1, 2]. When sustained, this pattern leads to progressive left ventricular remodeling and systolic dysfunction [3, 4]. LBBAP targets the conduction system distal to common sites of pacing-induced or coexisting conduction delay, restoring near-physiological ventricular activation and narrowing the paced QRS by approximately 50 ms in this analysis. The resulting electrical resynchronization was accompanied by consistent improvement in LVEF, reduced left ventricular dimensions, and symptomatic benefit across all included studies, supporting the mechanistic link between physiological activation and ventricular recovery. This degree of functional recovery approached that reported following conventional cardiac resynchronization therapy for pacing-induced cardiomyopathy [25, 26]. The magnitude of recovery appeared greater in cohorts with longer follow-up, which is consistent with the expected time course of reverse remodeling. The benefit extended to patients with severe PICM and a baseline LVEF less than 40%, where approximately three-quarters met the criteria for echocardiographic response, suggesting that even advanced ventricular dysfunction remains amenable to rescue with LBBAP upgrades. Structural remodeling also improved, with an approximately 4 mm reduction in the LVEDD, although the ventricular dimensions did not fully return to the pre-RVP baseline in studies that reported this comparison [20], suggesting that prolonged RVP may result in irreversible structural changes.

### Context within the existing literature

The concurrent meta-analysis by Jin et al. pooled 20 studies and 821 patients undergoing either His bundle or LBBAP upgrades and framed its conclusions at the level of conduction system pacing as a whole [27]. Our analysis differs in three deliberate respects. First, we restricted inclusion to LBBAP, excluding His bundle pacing and deep septal/left ventricular septal pacing series without confirmed left bundle capture, thereby isolating the modality of contemporary clinical relevance rather than a composite that blends it with strategies of declining use. Second, we re-verified every included study against its primary publication and applied source-level corrections. Third, we interrogated the LBBAP-specific estimate with leave-one-out, change-score correlation (r) sensitivity, and small-study analyses that a pooled conduction-system synthesis does not perform at the LBBAP level. Reassuringly, despite these differences, our LBBAP-specific LVEF estimate (+ 11.72%) aligns closely with both the overall CSP estimate (11.7%) and the LBBAP subgroup (11.8%) reported by Jin et al., providing external validation of the magnitude of recovery while attributing it specifically to LBBAP. At the prevention stage, the LBBP-FAVOUR randomized trial demonstrated that de novo LBBAP reduced the combined outcome of all-cause mortality, heart failure hospitalization, and PICM development by approximately 69% compared with RVP in patients with an anticipated high pacing burden [10]. Although LBBP-FAVOUR addresses prevention rather than rescue of established PICM, the direction of effect aligns with the findings of this analysis and together describes a consistent physiological pacing benefit spanning both prevention and treatment. The paced QRS duration after LBBAP in the included studies, ranging from 114 to 135 ms, compares favorably with the 150 ms reported with RVP at 36 months in LBBP-FAVOUR, and the larger absolute LVEF gain observed in the rescue setting likely reflects both greater room for improvement and the potential for reversible cardiac remodeling in established PICM. Our single-arm findings are likewise consistent with additional recent LBBAP-upgrade cohorts reported in pacing-induced ventricular dysfunction [28].

### LBBAP versus biventricular pacing

A key contribution of this analysis is a pooled head-to-head comparison of LBBAP and BiVP upgrade strategies in established PICM, an area in which few pooled analyses have focused specifically on LBBAP. Across two comparative studies enrolling 170 patients, LBBAP was associated with approximately 4.6% points greater LVEF recovery than BiVP, with a consistently narrower paced QRS duration and higher echocardiographic response rates [18, 29]. LBBAP recruits the native conduction system distal to the site of block, achieving a degree of electrical resynchronization that epicardial coronary sinus pacing cannot reliably replicate. He et al. further demonstrated that the LVEF advantage was more pronounced among patients who achieved true left bundle branch capture than among those with left ventricular septal pacing (+ 10.62% versus + 6.47%), reinforcing the physiological importance of direct conduction system recruitment [18]. Clinical event rates were not significantly different between the LBBAP and BiVP arms in the one study that reported this outcome, although that comparison was substantially underpowered [18]. These findings should be interpreted as hypothesis-generating. The comparative evidence base rests on two observational studies, and an I² of 0% across only two studies provides no reliable information about true between-study homogeneity. Adequately powered randomized data comparing LBBAP and BiVP upgrades in established PICM are needed before definitive conclusions can be drawn. These observations are directionally consistent with recent observational comparisons of conduction-system or LBBAP versus biventricular upgrades in PICM, which report favorable or comparable outcomes without randomized confirmation [30–32].

### Clinical implications

The findings of this analysis have two practical implications for clinicians managing patients with established or anticipated PICM. First, in patients who have already developed PICM, the LBBAP upgrade represents an effective rescue strategy and warrants consideration alongside the BiVP upgrade in centers with the requisite implantation expertise [33]. The high procedural success rate of 96.4% and the low complication rate of 1.8% support the feasibility of this approach in appropriately selected patients. Second, in patients with anticipated high pacing burden and any cardiac vulnerability factor, the convergence of evidence from this analysis and the LBBP-FAVOUR trial supports the consideration of de novo LBBAP over conventional RVP at the time of initial implantation, with the goal of preventing ventricular dysfunction before it develops [10]. Current guidelines already recognize conduction system pacing as a reasonable alternative to RVP in patients at risk of PICM [5, 6]. The present findings add to the evidence base supporting this recommendation, particularly in centers where LBBAP expertise is available. These implications should be interpreted in the context of the observational evidence base and GRADE certainty ratings ranging from low to very low. They are not intended to supplant current guideline recommendations but rather to inform clinical decision-making where randomized data remain unavailable.

### Limitations

Several limitations of this analysis warrant consideration. All included studies were nonrandomized observational designs, which precludes causal inference and leaves selection bias and residual confounding as concerns that cannot be fully excluded. GRADE certainty was rated low to very low across all outcomes, reflecting this fundamental limitation of the evidence base. The follow-up duration was short across the included studies, ranging from 4.7 to 27.3 months, which is sufficient for echocardiographic and electrical outcomes but inadequate for assessing hard clinical endpoints such as mortality and heart failure hospitalizations. The comparative analysis is based on only two studies enrolling 170 patients, which is insufficient to draw definitive conclusions about the superiority of LBBAP over BiVP, particularly for clinical outcomes. Five of the nine studies originated from Chinese centers, and some patients may have been included in more than one cohort. If patient overlap existed, the effective sample size would be smaller than reported, and the pooled estimates may appear more precise than the underlying data justify. PICM definitions varied modestly across studies, with differences in the LVEF decline threshold and minimum RVP burden needed, limiting strict comparability across cohorts. The LVEF sensitivity analysis across *r* = 0.2 to 0.8 confirmed the robustness of the primary estimate, although the change-score standard deviation was imputed for four of nine studies using an assumed within-subject correlation. The review protocol was registered in PROSPERO after the search was completed, which represents a methodological limitation, as retrospective registration does not prevent selective outcome reporting. Meta-regression was not performed because the number of studies was insufficient for reliable covariate adjustment. The substantial heterogeneity observed for QRS reduction and NYHA improvement likely reflects clinical variability across studies, including differing PICM definitions, baseline LVEF and RVP burden, follow-up duration, the criteria used to confirm left bundle branch capture, and operator experience and learning curve. Definitions of procedural success were not uniform across studies, and the extent of AV-delay optimization and post-upgrade device programming was inconsistently reported. Improvement in single-arm cohorts may additionally be influenced by regression to the mean, medication optimization, and patient selection.

## Conclusion

The available observational evidence supports LBBAP as an effective upgrade strategy for established PICM, but the observational nature of all included studies means that randomized confirmation remains the critical missing piece. An adequately powered randomized controlled trial comparing LBBAP with BiVP upgrades in patients with established PICM, with prespecified hard clinical endpoints and a minimum follow-up of 24 months, is needed to establish guideline-level evidence for this approach. Until such evidence is available, the findings of this analysis support LBBAP upgrades as a feasible and physiologically rational strategy for patients with PICM, with device-related complications uncommon in short-term observational data, to be considered alongside BiVP in centers with appropriate expertise.

## Supplementary Information

Supplementary Material 1.

Supplementary Material 2.

Supplementary Material 3.

Supplementary Material 4.

## Data Availability

All data supporting the findings of this study are available within the published article. The studies analyzed in this systematic review and meta-analysis are publicly available peer-reviewed publications cited in the reference list. No new primary data were generated.
