# Supplementary material for: Upgrading right ventricular pacing to left bundle branch area pacing in pacemaker-induced cardiomyopathy: a systematic review and meta-analysis
Source: BMC Cardiovasc Disord. 2026 Aug 14;26:1062. doi: 10.1186/s12872-026-06430-3 (PMC13753369; doi:10.1186/s12872-026-06430-3)
Supplement: Supplementary file 1 [file 12872_2026_6430_MOESM1_ESM.docx]

**Supplemental Table S7. Study-level PICM definitions, right ventricular pacing burden, and LBBAP capture characteristics**

| **Study** | **PICM definition (as reported)** | **RVP burden (%)** | **LBBAP capture confirmation** | **True LBBP vs LVSP** |
| --- | --- | --- | --- | --- |
| **Rademakers 2022** | ≥10% LVEF decline to <50%; RVP >20% | 99 ± 1.5 | Paced V1 RBBB conduction-delay/block morphology + short, stable LV activation time (no validated cutoff; mean LVAT 81 ms) | Not reported (LBBAP as a group) |
| **Ye 2021** | ≥10% LVEF decline to <50%; infranodal AVB; RVP >40% | 99 ± 1.5 | LBBP confirmed and differentiated from LVSP by previously published criteria | 19 LBBP / 0 LVSP (1 LVSP fixation failure excluded) |
| **Qian 2021** | ≥10% LVEF decline to <50%; RVP >20% | 96.5 ± 7.0 | Selective LBBP: V1 “M”/RBBB morphology, discrete LBB potential, V5/V6 R-wave peak time | Not reported (LBBAP as a group) |
| **Perea-Armijo 2025** | Severe PICM: post-RVP LVEF ≤40%; pre-implant LVEF >50%; RVP ≥20% | 98.5 [97–99] | V6-RWPT <100 ms; V6–V1 interpeak >33 ms; NS→S-LBBP transition, or transition to LVSP with V6-RWPT prolongation ≥15 ms | Capture types defined (S-/NS-LBBP, LVSP); distribution not separately reported (both grouped as LBBAP) |
| **Pei-pei M 2024** | Post-RVP LVEF ≤40%; pre-implant LVEF ≥50%; RVP >40% | ~93 | Abrupt decrease in Stim-LV activation time (>10 and <75 ms) + Qr/qR/rSR′ morphology in V1 | 13 LBBP / 0 LVSP (all LBBP; comparator HBP) |
| **Shan 2023** | ≥10% LVEF decline to <50%; RVP >20% | 97.9 ± 3.7 | V1 RBBB pattern + LBB potential + abrupt, constant shortest peak LV activation time | Not reported (LBBAP as a group) |
| **He 2026** | ≥10% LVEF decline to <50%; RVP >40% | 92.9 ± 13.6 | True LBBP distinguished from LVSP by standard capture criteria (V1 morphology, R-wave peak time) | 30 LBBP / 10 LVSP |
| **Shah 2026** | LVEF decline attributable to RVP; RVP >80% | 95.4 ± 10.4 | V5/V6 R-wave peak time measured at 1.0 and 5.0 V outputs | Not reported (LBBAP as a group) |
| **Moraleda-Salas 2025** | ≥10% LVEF decline after RVP | ~100 | Standard LBBAP capture criteria (not separately detailed; UHF-ECG used for dyssynchrony) | Not reported |

*PICM definitions, RVP burden, and capture-confirmation criteria are reproduced from each primary publication. “Not reported” indicates the study reported outcomes for LBBAP as a single group without separately quantifying true left bundle branch pacing (LBBP) versus left ventricular septal pacing (LVSP). Only He et al. (30 LBBP / 10 LVSP) reported an explicit split; Ye et al. and Pei-pei M et al. included only successful true-LBBP patients; Perea-Armijo et al. defined capture types but grouped them as LBBAP for analysis. RVP = right ventricular pacing; RWPT = R-wave peak time; S-/NS-LBBP = selective/non-selective LBBP; LVAT = left ventricular activation time; AVB = atrioventricular block.*
