# Supplementary material for: Upgrading right ventricular pacing to left bundle branch area pacing in pacemaker-induced cardiomyopathy: a systematic review and meta-analysis
Source: BMC Cardiovasc Disord. 2026 Aug 14;26:1062. doi: 10.1186/s12872-026-06430-3 (PMC13753369; doi:10.1186/s12872-026-06430-3)
Supplement: Supplementary file 2 [file 12872_2026_6430_MOESM2_ESM.docx]

# Supplemental Text S1. Literature Search Strategy

*Upgrading Right Ventricular Pacing to Left Bundle Branch Area Pacing in Pacemaker-Induced Cardiomyopathy: A Systematic Review and Meta-Analysis.*

**PROSPERO registration:** CRD420261381943

**Reporting standard:** PRISMA 2020

**Search period:** From database inception to 31 March 2026

A systematic search was performed across four electronic databases — PubMed (MEDLINE), Embase, the Cochrane Library (including CENTRAL), and Scopus — supplemented by backward citation searching, trial-registry searching, and gray-literature screening. Search terms were combined across two conceptual domains: (1) pacing modality (left bundle branch area pacing and related conduction-system / physiologic pacing terms) AND (2) clinical context (right ventricular pacing, device upgrade, and pacing- or pacemaker-induced cardiomyopathy). No language restriction was applied. The exact strings run in each database are reproduced verbatim below.

| **Database** | **Interface / field** | **Records identified** | **Date range searched** |
| --- | --- | --- | --- |
| PubMed (MEDLINE) | pubmed.gov — All Fields | 612 | Inception – 31 Mar 2026 |
| Embase | embase.com — ti,ab | 521 | Inception – 31 Mar 2026 |
| Cochrane Library (incl. CENTRAL) | Wiley — All Text; Trials + Reviews | 187 | Inception – 31 Mar 2026 |
| Scopus | scopus.com — TITLE-ABS-KEY | 527 | Inception – 31 Mar 2026 |
| Other sources (hand-search) | Registries, proceedings, reference lists | 24 | Inception – 31 Mar 2026 |
| **Total identified** |  | **1,871** |  |

*Counts above reflect the search through the 31 March 2026 review cutoff. Verification re-runs performed after the cutoff return marginally higher counts, consistent with ongoing database indexing.*

## Database search strings

**1. PubMed (MEDLINE) — pubmed.gov**

**Field:** All Fields. Limits: inception – 31 March 2026.

("left bundle branch pacing" OR "left bundle branch area pacing" OR LBBP OR LBBAP OR "conduction system pacing" OR "His bundle pacing" OR "His-Purkinje" OR "physiologic pacing" OR "physiological pacing") AND ("right ventricular pacing" OR "RV pacing" OR "pacemaker upgrade" OR "device upgrade" OR "pacing-induced cardiomyopathy" OR "pacemaker-induced cardiomyopathy" OR PICM)

**2. Embase — embase.com (Elsevier)**

**Interface:** Advanced Search. Field: title/abstract (:ti,ab).

('left bundle branch pacing' OR 'left bundle branch area pacing' OR LBBP OR LBBAP OR 'conduction system pacing' OR 'his bundle pacing' OR 'his purkinje' OR 'physiologic pacing' OR 'physiological pacing' OR 'left ventricular septal pacing' OR 'deep septal pacing'):ti,ab AND ('pacemaker upgrade' OR 'device upgrade' OR 'pacing-induced cardiomyopathy' OR 'pacing induced cardiomyopathy' OR 'pacemaker-induced cardiomyopathy' OR 'pacemaker induced cardiomyopathy' OR PICM OR 'right ventricular apical pacing' OR 'rv pacing'):ti,ab

**3. Cochrane Library (including CENTRAL) — Wiley**

**Field:** All Text. Counted across the Trials and Cochrane Reviews tabs.

("left bundle branch pacing" OR "left bundle branch area pacing" OR LBBP OR LBBAP OR "conduction system pacing" OR "His bundle pacing" OR "His Purkinje" OR "physiologic pacing" OR "physiological pacing") AND ("right ventricular pacing" OR "pacemaker upgrade" OR "device upgrade" OR "pacing-induced cardiomyopathy" OR "pacing induced cardiomyopathy" OR PICM OR upgrade OR "cardiac resynchronization" OR "cardiac resynchronisation")

**4. Scopus — scopus.com (Elsevier)**

**Interface:** Advanced document search. Field: TITLE-ABS-KEY.

TITLE-ABS-KEY("left bundle branch pacing" OR "left bundle branch area pacing" OR LBBP OR LBBAP OR "conduction system pacing" OR "His bundle pacing" OR "His Purkinje" OR "physiologic pacing" OR "physiological pacing" OR "left ventricular septal pacing" OR "deep septal pacing") AND TITLE-ABS-KEY("right ventricular pacing" OR "RV pacing" OR "pacemaker upgrade" OR "device upgrade" OR "pacing-induced cardiomyopathy" OR "pacing induced cardiomyopathy" OR "pacemaker-induced cardiomyopathy" OR "pacemaker induced cardiomyopathy" OR PICM)

## Other sources (hand-searching)

Conference proceedings of the Heart Rhythm Society, the European Society of Cardiology, and the American College of Cardiology (2020–2025); trial registries (ClinicalTrials.gov and the ISRCTN registry); and backward citation (reference-list) screening of included studies and relevant reviews. These sources contributed 24 additional records.

## De-duplication and screening

The 1,871 identified records (1,847 from databases plus 24 from other sources) were reduced to 1,391 after removal of 480 duplicates. Title/abstract screening excluded 1,300 records; 91 full-text articles were assessed for eligibility; 82 were excluded with reasons (detailed in the PRISMA flow diagram, Figure S6); and 9 studies were included in the qualitative and quantitative synthesis.

*Note. All databases were searched from inception to 31 March 2026; the record counts shown correspond to this search period. Verification re-runs performed after the cutoff return marginally higher counts owing to ongoing database indexing.*
