# Supplementary material for: Upgrading right ventricular pacing to left bundle branch area pacing in pacemaker-induced cardiomyopathy: a systematic review and meta-analysis
Source: BMC Cardiovasc Disord. 2026 Aug 14;26:1062. doi: 10.1186/s12872-026-06430-3 (PMC13753369; doi:10.1186/s12872-026-06430-3)
Supplement: Supplementary file 3 [file 12872_2026_6430_MOESM3_ESM.docx]

**PRISMA 2020 Checklist**

*Upgrading Right Ventricular Pacing to Left Bundle Branch Area Pacing in Pacemaker-Induced Cardiomyopathy: A Systematic Review and Meta-Analysis*

**Reference:** Page MJ, McKenzie JE, Bossuyt PM, et al. The PRISMA 2020 statement: an updated guideline for reporting systematic reviews. BMJ 2021;372:n71. https://doi.org/10.1136/bmj.n71

| **Section / Topic** | **Item #** | **Checklist Item** | **Location Reported** |
| --- | --- | --- | --- |
| **TITLE** | 1 | Identify the report as a systematic review. | Title page |
| **ABSTRACT** | 2 | See PRISMA 2020 for Abstracts checklist. | Abstract (structured Background / Methods / Results / Conclusion) |
| **INTRODUCTION** | 3 | Describe the rationale for the review in the context of existing knowledge. | Introduction, paragraphs 1–2 |
|  | 4 | Provide an explicit statement of the objective(s) or question(s) the review addresses. | Introduction, paragraph 3 (final paragraph) |
| **METHODS** | 5 | Specify the inclusion and exclusion criteria for the review and how studies were grouped for the syntheses. | Methods – PICO Framework and Eligibility Criteria |
|  | 6 | Specify all databases, registers, websites, organisations, reference lists and other sources searched or consulted to identify studies. Specify the date when each source was last searched or consulted. | Methods – Search Strategy (PubMed, Embase, Cochrane, Scopus; through 31 March 2026) |
|  | 7 | Present the full search strategies for all databases, registers and websites, including any filters and limits used. | Methods – Search Strategy; full PubMed strategy in Supplemental Text S1 |
|  | 8 | Specify the methods used to decide whether a study met the inclusion criteria of the review, including how many reviewers screened each record and each report retrieved, whether they worked independently, and if applicable, details of automation tools used in the process. | Methods – Data Extraction and Risk of Bias (two independent reviewers; consensus for discrepancies) |
|  | 9 | Specify the methods used to collect data from reports, including how many reviewers collected data from each report, whether they worked independently, any processes for obtaining or confirming data from study investigators, and if applicable, details of automation tools used in the process. | Methods – Data Extraction and Risk of Bias |
|  | 10a | List and define all outcomes for which data were sought. Specify whether all results that were compatible with each outcome domain in each study were sought (e.g. for all measures, time points, analyses), and if not, the methods used to decide which results to collect. | Methods – PICO Framework (Outcome O); Methods – Statistical Analysis |
|  | 10b | List and define all other variables for which data were sought (e.g. participant and intervention characteristics, funding sources). Describe any assumptions made about any missing or unclear information. | Methods – Data Extraction and Risk of Bias; Methods – Statistical Analysis (r=0.5 assumption for change-score SD imputation) |
|  | 11 | Specify the methods used to assess risk of bias in the included studies, including details of the tool(s) used, how many reviewers assessed each study and whether they worked independently, and if applicable, details of automation tools used in the process. | Methods – Data Extraction and Risk of Bias (MINORS tool) |
|  | 12 | Specify for each outcome the effect measure(s) (e.g. risk ratio, mean difference) used in the synthesis or presentation of results. | Methods – Statistical Analysis (MD with 95% CI; proportion via Freeman-Tukey) |
|  | 13a | Describe the processes used to decide which studies were eligible for each synthesis (e.g. tabulating the study intervention characteristics and comparing against the planned groups for each synthesis (item #5)). | Methods – Statistical Analysis; Results – Characteristics of Included Studies |
|  | 13b | Describe any methods required to prepare the data for presentation or synthesis, such as handling of missing summary statistics, or data conversions. | Methods – Statistical Analysis (SDchange imputation from pre/post; Wan IQR conversion for Perea-Armijo) |
|  | 13c | Describe any methods used to tabulate or visually display results of individual studies and syntheses. | Methods – Statistical Analysis; Tables 1–3; Figure 1 (PRISMA); Supplemental Figures S1–S5 |
|  | 13d | Describe any methods used to synthesise results and provide a rationale for the choice(s). If meta-analysis was performed, describe the model(s), method(s) to identify the presence and extent of statistical heterogeneity, and software package(s) used. | Methods – Statistical Analysis (REML; Python 3.12 with NumPy/SciPy; metafor Berkey 1995 validation) |
|  | 13e | Describe any methods used to explore possible causes of heterogeneity among study results (e.g. subgroup analysis, meta-regression). | Methods – Statistical Analysis (leave-one-out; sensitivity to r; sensitivity excluding Moraleda-Salas) |
|  | 13f | Describe any sensitivity analyses conducted to assess robustness of the synthesised results. | Methods – Statistical Analysis; Results – Sensitivity Analysis; Supplemental Tables S1–S4 |
|  | 14 | Describe any methods used to assess risk of bias due to missing results in a synthesis (arising from reporting biases). | Methods – Statistical Analysis (Begg-Mazumdar; Egger’s regression) |
|  | 15 | Describe any methods used to assess certainty (or confidence) in the body of evidence for an outcome. | Methods – Data Extraction and Risk of Bias (GRADE framework); Table 3 |
| **RESULTS** | 16a | Describe the results of the search and selection process, from the number of records identified in the search to the number of studies included in the review, ideally using a flow diagram. | Results – Study Selection; Figure 1 (PRISMA 2020 flow diagram) |
|  | 16b | Cite studies that might appear to meet the inclusion criteria, but which were excluded, and explain why they were excluded. | Results – Study Selection (Jin 2026, Morcé 2025, Yang 2021, Wang 2023, others) |
|  | 17 | Cite each included study and present its characteristics. | Table 1; Results – Characteristics of Included Studies |
|  | 18 | Present assessments of risk of bias for each included study. | MINORS scores in Results (range 11–22); Table 1 |
|  | 19 | For all outcomes, present, for each study: (a) summary statistics for each group (where appropriate) and (b) an effect estimate and its precision (e.g. confidence/credible interval), ideally using structured tables or plots. | Table 1 (study-level data); Supplemental Table S6 (raw data); Supplemental Figures S1–S4 (forest plots) |
|  | 20a | For each synthesis, briefly summarise the characteristics and risk of bias among contributing studies. | Results – Characteristics of Included Studies; Table 1 |
|  | 20b | Present results of all statistical syntheses conducted. If meta-analysis was done, present for each the summary estimate and its precision (e.g. confidence/credible interval) and measures of statistical heterogeneity. If comparing groups, describe the direction of the effect. | Table 2; Results – Primary Outcomes, Secondary Outcomes, Comparative Analysis |
|  | 20c | Present results of all investigations of possible causes of heterogeneity among study results. | Results – Sensitivity Analysis; Supplemental Tables S1–S4 |
|  | 20d | Present results of all sensitivity analyses conducted to assess the robustness of the synthesised results. | Results – Sensitivity Analysis: Excluding Moraleda-Salas; Supplemental Tables S1, S4 |
|  | 21 | Present assessments of risk of bias due to missing results (arising from reporting biases) for each synthesis assessed. | Results – Publication Bias and Small-Study Effects; Supplemental Figure S5 |
|  | 22 | Present assessments of certainty (or confidence) in the body of evidence for each outcome assessed. | Table 3 (GRADE certainty summary) |
| **DISCUSSION** | 23a | Provide a general interpretation of the results in the context of other evidence. | Discussion – opening paragraph; Why LBBAP Reverses PICM; LBBAP versus BiVP Upgrade in PICM; Concordance with the LBBP-FAVOUR Randomised Trial; Concordance with Other Systematic Reviews |
|  | 23b | Discuss any limitations of the evidence included in the review. | Discussion – Limitations |
|  | 23c | Discuss any limitations of the review processes used. | Discussion – Limitations |
|  | 23d | Discuss implications of the results for practice, policy, and future research. | Discussion – Concordance with LBBP-FAVOUR (clinical implications); Conclusion (need for RCT of LBBAP vs BiVP upgrade) |
| **OTHER INFORMATION** | 24a | Provide registration information for the review, including the register name and registration number, or state that the review was not registered. | Title page (PROSPERO CRD420261381943); Methods – Protocol Registration |
|  | 24b | Indicate where the review protocol can be accessed, or state that a protocol was not prepared. | Methods – Protocol Registration (publicly accessible via PROSPERO database) |
|  | 24c | Describe and explain any amendments to information provided at registration or in the protocol. | Methods – Protocol Registration. Four deviations disclosed per item 24c: (1) REML used as the primary heterogeneity estimator instead of DerSimonian–Laird; (2) MINORS selected for risk-of-bias assessment given mixed comparative/non-comparative designs; (3) three prespecified subgroup analyses not performed because the final study count (k=9) was insufficient; (4) two studies retained with follow-up slightly outside the registered 6–24 month window. None altered the registered PICO, primary outcomes, or eligibility criteria. |
|  | 25 | Describe sources of financial or non-financial support for the review, and the role of the funders or sponsors in the review. | Statements and Declarations – Funding (none received) |
|  | 26 | Declare any competing interests of review authors. | Statements and Declarations – Competing Interests (none) |
|  | 27 | Report which of the following are publicly available and where they can be found: template data collection forms; data extracted from included studies; data used for all analyses; analytic code; any other materials used in the review. | Statements and Declarations – Data availability (data extraction sheet provided as Supplementary Information) |
