# Supplementary material for: Upgrading right ventricular pacing to left bundle branch area pacing in pacemaker-induced cardiomyopathy: a systematic review and meta-analysis
Source: BMC Cardiovasc Disord. 2026 Aug 14;26:1062. doi: 10.1186/s12872-026-06430-3 (PMC13753369; doi:10.1186/s12872-026-06430-3)
Supplement: Supplementary file 4 [file 12872_2026_6430_MOESM4_ESM.docx]

**Supplemental Appendix**

*Upgrading Right Ventricular Pacing to Left Bundle Branch Area Pacing in Pacemaker-Induced Cardiomyopathy: A Systematic Review and Meta-Analysis*

This Supplemental Appendix accompanies the primary manuscript and provides: (i) leave-one-out sensitivity analyses for the three pooled continuous outcomes (Tables S1–S3); (ii) the within-subject-correlation (r) sensitivity analysis for the primary LVEF estimate (Table S4); (iii) risk-of-bias scoring per the Methodological Index for Non-Randomized Studies (Table S5); (iv) the study-level raw data underpinning all pooled estimates (Table S6); and (v) descriptive captions for Figures S1–S6.

**Estimator note.** All pooled effect estimates are reported using restricted maximum likelihood (REML) as the primary random-effects model, consistent with current Cochrane guidance and the metafor package default. Each table additionally reports the DerSimonian–Laird (DL) estimator as a parallel cross-check, as pre-specified in the Methods. Across every outcome and every leave-one-out iteration, REML and DL estimates agree to within rounding, supporting the robustness of the pooled findings to the choice of variance estimator.

# **Contents**

Table S1. Leave-One-Out Sensitivity Analysis — LVEF Improvement

Table S2. Leave-One-Out Sensitivity Analysis — QRS Duration Reduction

Table S3. Leave-One-Out Sensitivity Analysis — NYHA Class Improvement

Table S4. Sensitivity to Within-Subject Correlation Assumption (r) — LVEF

Table S5. Risk of Bias Assessment (MINORS) for Included Studies

Table S6. Study-Level Raw Data Used in Meta-Analysis

Figure S1. Funnel Plot — LVEF Improvement

Figure S2. Forest Plot — LVEF Improvement (Primary Outcome)

Figure S3. Leave-One-Out Sensitivity Plot — LVEF Improvement

Figure S4. Sensitivity to r Assumption — LVEF Improvement

Figure S5. Comparative Forest Plot — LBBAP vs BiVP (LVEF Improvement)

Figure S6. PRISMA 2020 Flow Diagram

## **Table S1. Leave-One-Out Sensitivity Analysis — LVEF Improvement**

Each row shows the pooled mean difference (MD) in LVEF improvement when the named study is excluded. REML is the primary estimator; DerSimonian–Laird (DL) is reported as a parallel cross-check. Full-model estimate (k=9): REML +11.72% (+9.85, +13.59), I²=65.1%; DL +11.72% (+9.89, +13.56), I²=63.5%. All leave-one-out estimates were statistically significant (p<0.001).

| **Study Excluded** | **k** | **N** | **REML MD (95% CI)** | **REML I²** | **DL MD (95% CI)** | **DL I²** |
| --- | --- | --- | --- | --- | --- | --- |
| Rademakers et al. 2022 | 8 | 222 | +11.34% (+9.36, +13.32) | 64.1% | +11.34% (+9.42, +13.27) | 61.8% |
| Ye et al. 2021 | 8 | 223 | +11.39% (+9.47, +13.31) | 65.6% | +11.40% (+9.52, +13.27) | 63.8% |
| Qian et al. 2021 | 8 | 229 | +12.11% (+10.22, +14.00) | 63.5% | +12.11% (+10.22, +14.00) | 63.5% |
| Perea-Armijo et al. 2025 | 8 | 217 | +11.49% (+9.46, +13.52) | 68.8% | +11.50% (+9.55, +13.46) | 66.3% |
| Pei-pei M et al. 2024 | 8 | 229 | +11.94% (+9.73, +14.14) | 63.8% | +11.93% (+9.70, +14.17) | 64.8% |
| Shan et al. 2023 | 8 | 204 | +11.22% (+9.37, +13.06) | 55.7% | +11.21% (+9.41, +13.00) | 53.0% |
| He et al. 2026 | 8 | 202 | +12.09% (+10.05, +14.13) | 62.5% | +12.09% (+10.04, +14.14) | 62.7% |
| Shah et al. 2026 | 8 | 178 | +11.79% (+9.69, +13.90) | 70.6% | +11.80% (+9.78, +13.81) | 68.0% |
| Moraleda-Salas et al. 2025 | 8 | 232 | +12.06% (+10.19, +13.93) | 64.8% | +12.05% (+10.21, +13.90) | 63.9% |

Range across iterations: REML +11.22% to +12.11%; DL +11.21% to +12.11%. No single study disproportionately drives the pooled estimate.

## **Table S2. Leave-One-Out Sensitivity Analysis — QRS Duration Reduction**

Each row shows the pooled MD for QRS reduction when the named study is excluded. Full-model estimate (k=9): REML -51.83 ms (-57.68, -45.99), I²=83.2%; DL -51.84 ms (-57.82, -45.86), I²=83.9%. The persistently high I² across all iterations confirms heterogeneity is distributed across the full study set rather than driven by a single outlier.

| **Study Excluded** | **k** | **N** | **REML MD (95% CI)** | **REML I²** | **DL MD (95% CI)** | **DL I²** |
| --- | --- | --- | --- | --- | --- | --- |
| Rademakers et al. 2022 | 8 | 222 | -50.44 ms (-56.28, -44.59) | 81.3% | -50.44 ms (-56.41, -44.48) | 82.0% |
| Ye et al. 2021 | 8 | 223 | -51.44 ms (-57.96, -44.91) | 85.4% | -51.44 ms (-58.00, -44.87) | 85.6% |
| Qian et al. 2021 | 8 | 229 | -51.40 ms (-57.91, -44.88) | 85.3% | -51.40 ms (-57.97, -44.83) | 85.5% |
| Perea-Armijo et al. 2025 | 8 | 217 | -51.57 ms (-58.18, -44.97) | 85.2% | -51.58 ms (-58.29, -44.86) | 85.7% |
| Pei-pei M et al. 2024 | 8 | 229 | -51.26 ms (-57.79, -44.73) | 84.3% | -51.26 ms (-57.92, -44.61) | 84.9% |
| Shan et al. 2023 | 8 | 204 | -51.26 ms (-57.68, -44.83) | 85.2% | -51.26 ms (-57.73, -44.79) | 85.4% |
| He et al. 2026 | 8 | 202 | -51.83 ms (-58.52, -45.14) | 84.5% | -51.84 ms (-58.83, -44.84) | 85.9% |
| Shah et al. 2026 | 8 | 178 | -53.67 ms (-58.96, -48.38) | 74.1% | -53.68 ms (-58.67, -48.70) | 70.8% |
| Moraleda-Salas et al. 2025 | 8 | 232 | -53.64 ms (-58.80, -48.48) | 76.7% | -53.72 ms (-59.45, -47.99) | 81.2% |

Range across iterations: REML -53.68 to -50.46 ms. All p<0.001.

## **Table S3. Leave-One-Out Sensitivity Analysis — NYHA Class Improvement**

Each row shows the pooled MD for NYHA class improvement when the named study is excluded. Full-model estimate (k=7, two studies did not report NYHA): REML -0.980 (-1.204, -0.757), I²=85.4%; DL -0.981 (-1.196, -0.765), I²=84.3%. All p<0.001.

| **Study Excluded** | **k** | **N** | **REML MD (95% CI)** | **REML I²** | **DL MD (95% CI)** | **DL I²** |
| --- | --- | --- | --- | --- | --- | --- |
| Rademakers et al. 2022 | 6 | 184 | -0.906 (-1.105, -0.706) | 77.4% | -0.906 (-1.090, -0.722) | 73.4% |
| Ye et al. 2021 | 6 | 185 | -1.025 (-1.266, -0.785) | 86.1% | -1.025 (-1.258, -0.793) | 85.1% |
| Qian et al. 2021 | 6 | 191 | -1.004 (-1.254, -0.754) | 88.2% | -1.004 (-1.239, -0.769) | 86.6% |
| Shan et al. 2023 | 6 | 166 | -0.916 (-1.130, -0.701) | 82.4% | -0.916 (-1.130, -0.701) | 82.4% |
| He et al. 2026 | 6 | 164 | -1.018 (-1.268, -0.768) | 86.3% | -1.018 (-1.259, -0.778) | 85.2% |
| Shah et al. 2026 | 6 | 140 | -0.982 (-1.250, -0.714) | 86.3% | -0.981 (-1.255, -0.708) | 86.9% |
| Moraleda-Salas et al. 2025 | 6 | 194 | -1.013 (-1.268, -0.757) | 86.0% | -1.013 (-1.261, -0.765) | 85.0% |

Range across iterations: REML -1.025 to -0.905.

## **Table S4. Sensitivity to Within-Subject Correlation Assumption (r) — LVEF Improvement**

REML and DL pooled MDs for LVEF improvement under seven values of the assumed within-subject correlation (r) used to derive change-score SDs when not directly reported. Five of nine studies (Perea-Armijo, Pei-pei M, He, Shah, Moraleda-Salas) report direct change-score SDs that do not depend on r; the remaining four studies use SD_change_ = √(SD_pre_² + SD_post_² − 2r·SD_pre_·SD_post_) at the indicated r. The pooled estimate shifts by only 0.23 percentage points (range +11.59% to +11.82%), while the heterogeneity statistics shift visibly (I² 52%–77%, τ² 3.5–6.3), which is the expected signature of a robust pool.

| **r** | **REML MD (%)** | **REML 95% CI** | **REML I²** | **DL MD (%)** | **DL 95% CI** | **DL I²** |
| --- | --- | --- | --- | --- | --- | --- |
| 0.2 | +11.591 | (+9.81, +13.37) | 53.9% | +11.585 | (+9.83, +13.34) | 52.2% |
| 0.3 | +11.637 | (+9.82, +13.45) | 57.5% | +11.634 | (+9.86, +13.41) | 55.8% |
| 0.4 | +11.681 | (+9.84, +13.52) | 61.3% | +11.680 | (+9.88, +13.48) | 59.5% |
| 0.5 | +11.721 | (+9.85, +13.59) | 65.1% | +11.722 | (+9.89, +13.56) | 63.5% |
| 0.6 | +11.757 | (+9.85, +13.66) | 69.0% | +11.760 | (+9.89, +13.63) | 67.8% |
| 0.7 | +11.790 | (+9.85, +13.73) | 73.3% | +11.794 | (+9.89, +13.70) | 72.3% |
| 0.8 | +11.819 | (+9.85, +13.79) | 77.9% | +11.823 | (+9.88, +13.77) | 77.3% |

## **Table S5. Risk of Bias Assessment — Methodological Index for Non-Randomized Studies (MINORS)**

Non-comparative studies are scored out of 16 (high quality ≥ 12; moderate 9–11; low < 9). Comparative studies are scored out of 24 (high ≥ 18; moderate 14–17; low < 14). Two independent reviewers scored each study; disagreements were resolved by consensus.

| **Study** | **Type** | **Comparator** | **Score / Max** | **Quality** |
| --- | --- | --- | --- | --- |
| Rademakers et al. 2022 | Non-comparative | — | 14 / 16 | High |
| Ye et al. 2021 | Non-comparative | — | 12 / 16 | High |
| Qian et al. 2021 | Non-comparative | — | 14 / 16 | High |
| Perea-Armijo et al. 2025 | Non-comparative | — | 14 / 16 | High |
| Pei-pei M et al. 2024 | Comparative | HBP | 20 / 24 | High |
| Shan et al. 2023 | Non-comparative | — | 12 / 16 | High |
| He et al. 2026 | Comparative | BiVP | 22 / 24 | High |
| Shah et al. 2026 | Non-comparative | — | 13 / 16 | High |
| Moraleda-Salas et al. 2025 | Non-comparative | — | 11 / 16 | Moderate |

Median MINORS score: 13 / 16 across non-comparative studies; 21 / 24 across comparative studies. No study scored in the low-quality range.

## **Table S6. Study-Level Raw Data Used in Meta-Analysis**

All values are transcribed from published source papers. Where the change-score SD was not directly reported by the source paper, it was estimated from pre- and post-upgrade SDs using SD_change_ = √(SD_pre_² + SD_post_² − 2r·SD_pre_·SD_post_) with r = 0.5. Direct change-score SDs (marked "direct") were used when reported. All seven post-extraction audit corrections (including the Shan 2023 complication recount, 2 → 1) are reflected in the values below.

| **Study** | **N** | **LVEF pre** | **LVEF post** | **ΔLVEF** | **QRS pre** | **QRS post** | **ΔQRS** | **Δ SD source** |
| --- | --- | --- | --- | --- | --- | --- | --- | --- |
| Rademakers 2022 | 20 | 32.6 ± 6.0 | 47.0 ± 8.0 | +14.4 | 193 ± 18 | 130 ± 17 | −63 | Estimated (r=0.5) |
| Ye 2021 | 19 | 36.3 ± 6.5 | 51.9 ± 13.0 | +15.6 | 176.2 ± 21.4 | 120.9 ± 15.2 | −55.3 | Estimated (r=0.5) |
| Qian 2021 | 13 | 40.3 ± 5.2 | 48.1 ± 9.5 | +7.8 | 173.1 ± 17.7 | 117.5 ± 10.2 | −55.6 | Estimated (r=0.5) |
| Perea-Armijo 2025 | 25 | 31.83 ± 3.33 | 45.6 ± 12.44 | +14.0 | 180 ± 20 | 126 ± 16.3 | −54 | Direct (Wan-IQR) |
| Pei-pei M 2024 | 13 | — | — | +10.55 | 170.3 ± 13.3 | 114.0 ± 9.3 | −56.3 | Direct (paper, LBBP-arm) |
| Shan 2023 | 38 | 36.6 ± 7.2 | 51.3 ± 8.7 | +14.7 | 174.9 ± 33.4 | 117.9 ± 21.4 | −57 | Estimated (r=0.5) |
| He 2026 | 40 | 37.4 ± 6.9 | 47.0 ± 9.5 | +9.59 | 187.6 ± 17.3 | 135.6 ± 19.3 | −52 | Direct (paper) |
| Shah 2026 | 64 | 36.5 ± 8.8 | 47.5 ± 10.6 | +11.0 | 172.5 ± 21.9 | 133.4 ± 19.2 | −39.1 | Direct (paper) |
| Moraleda-Salas 2025 | 10 | 40.2 ± 8.0 | 46.8 ± 8.5 | +6.6 | 158.3 ± 15 | 123.3 ± 14 | −35 | Estimated (r=0.5) |

ΔLVEF SDs: studies marked "Estimated (r=0.5)" had change SDs derived via the Cochrane formula above; "Direct" studies report the change SD directly in the source paper, except Perea-Armijo (median[IQR] converted to mean±SD via Wan 2014). Procedural success per study: Rademakers 20/20; Ye 19/19; Qian 13/13; Perea-Armijo 21/25; Pei-pei M 13/13; Shan 34/38; He 40/40; Shah 64/64; Moraleda-Salas 10/10 → crude 96.7%; Freeman–Tukey pooled 96.4% (95% CI 92.2–99.3%). Complications per study: 0, 0, 0, 0, 0, 1, 2, 0, 0 → 3 events / 242 (crude 1.24%); Freeman–Tukey pooled 1.8% (95% CI 0.2–4.5%). The Shan 2023 count was corrected from 2 to 1 in May 2026 on reviewer audit: only the PICM-arm pocket hematoma counts toward the PICM-arm rate; the additional lead revision was in a non-PICM patient.

# **Supplementary Figures**

**Figure S1.** Funnel plot of standard error versus mean difference in LVEF improvement for the nine included single-arm LBBAP studies. The funnel shows 95% and 99% confidence interval boundaries. Begg–Mazumdar rank correlation: ρ = −0.050, p = 0.898. Egger’s regression intercept: +0.483, SE = 1.445, p = 0.748. Neither test detected statistically significant funnel-plot asymmetry. With k = 9 studies, both tests have limited statistical power; trim-and-fill was therefore not applied.

**Figure S2.** Forest plot of LVEF improvement for the nine included single-arm LBBAP upgrade studies. Square area is proportional to the random-effects weight assigned to each study. The pooled estimate is shown as a red diamond. REML random-effects model with r = 0.5 for SD imputation where direct change-score SDs were not reported. Pooled MD: +11.72% (95% CI +9.85 to +13.59%; I²=63.5%; τ²=4.75; p<0.001). DL cross-check: +11.72% (95% CI +9.89 to +13.56%).

**Figure S3.** Leave-one-out sensitivity analysis for LVEF improvement. Each diamond shows the REML pooled estimate when the named study is excluded. The red dashed line and shaded band show the full-model estimate and 95% CI for reference. Range across iterations: +11.22% to +12.11%. All p<0.001.

**Figure S4.** Sensitivity of pooled MD (left axis) and I² (right axis) to the assumed within-subject correlation r (range 0.2–0.8). The pooled MD is stable across all plausible r values (REML +11.59% to +11.82%), while I² increases from ~54% to ~78% as r increases. Five of nine studies (Perea-Armijo, Pei-pei M, He, Shah, Moraleda-Salas) report direct change-score SDs that do not depend on r, explaining the narrow point-estimate range.

**Figure S5.** Comparative forest plot of LVEF improvement, LBBAP versus BiVP upgrade, in the two studies providing direct head-to-head comparative data (He 2026; Huang 2025). REML pooled MD: +4.59% (95% CI +2.23 to +6.95) in favor of LBBAP. I² = 0% with k = 2 is statistically uninformative and reflects only that the two effect estimates do not differ; this analysis is hypothesis-generating, pending randomized data.

**Figure S6.** PRISMA 2020 flow diagram. 1,871 records identified across PubMed/MEDLINE (612), Embase (521), Cochrane Library (187), Scopus (527), and reference-list / conference hand-searching (24). After deduplication (480 duplicates removed), 1,391 records advanced to title/abstract screening; 1,300 excluded. Of 91 full-text articles assessed for eligibility, 82 were excluded with specific reasons recorded; 9 single-arm LBBAP studies (N = 242) were included in the quantitative synthesis; 2 comparative studies (N = 170) contributed the LBBAP-versus-BiVP head-to-head pool.
